# Non-homologous DNA increases gene disruption efficiency by altering DNA repair outcomes

**DOI:** 10.1038/ncomms12463

**Published:** 2016-08-17

**Authors:** C. D. Richardson, G. J. Ray, N. L. Bray, J. E. Corn

**Affiliations:** 1 Innovative Genomics Initiative, University of California, Berkeley 94720, USA; 2 Department of Molecular and Cell Biology, University of California, Berkeley, California 94720, USA

## Abstract

The Cas9 endonuclease can be targeted to genomic sequences by programming the sequence of an associated single guide RNA (sgRNA). For unknown reasons, the activity of these Cas9–sgRNA combinations varies widely at different genomic loci and in different cell types. Thus, disrupting genes in polyploid cell lines or when using poorly performing sgRNAs can require extensive downstream screening to identify homozygous clones. Here we find that non-homologous single-stranded DNA greatly stimulates Cas9-mediated gene disruption in the absence of homology-directed repair. This stimulation increases the frequency of clones with homozygous gene disruptions and rescues otherwise ineffective sgRNAs. The molecular outcome of enhanced gene disruption depends upon cellular context, stimulating deletion of genomic sequence or insertion of non-homologous DNA at the edited locus in a cell line specific manner. Non-homologous DNA appears to divert cells towards error-prone instead of error-free repair pathways, dramatically increasing the frequency of gene disruption.

## Abstract (web summary)

CRISPR-Cas9 mediated gene editing has begun to revolutionize molecular biology, but editing efficiencies can vary greatly between reagents. The authors show that the addition of single-stranded non-homologous DNA stimulates gene disruption by favouring error-prone DNA repair.

## Graphical abstract

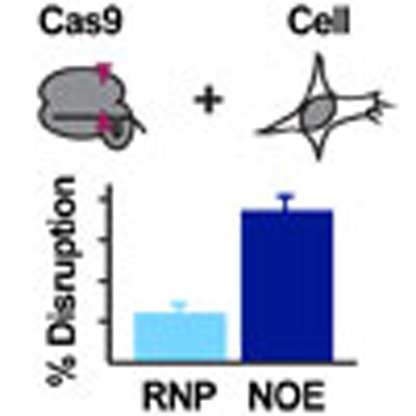

Programmable genetic disruption holds great promise for the investigation of gene function and translational potential for the treatment of genetic disease. Gene knockouts are commonly generated by introducing a site specific double strand break (DSB) within the gene of interest and screening for clones in which one or more alleles have been repaired in an error-prone fashion to disrupt the open reading frame^1^. The efficiency of this process is limited by the number of clones that must be screened to find the interruption, which is itself a product of the frequency of genome cutting and the frequency of disruptive repair events. The programmable Cas9 nuclease, which relies upon a targeting single guide RNA (sgRNA), has recently emerged as a popular tool for gene disruption due to its relative ease of use^2^. But Cas9–sgRNA combinations vary greatly in apparent cellular activity, from completely inactive to nearly 100% efficient, which can complicate experiments in which functional concerns restrict the genomic location to be targeted^3^,^4^,^5^,^6^. This variable activity has been attributed to differences in Cas9's ability to use sgRNAs of various sequences^7^,^8^, but differences in the activity of a given sgRNA between cell lines and organisms suggests that Cas9 introduction efficiency and location- or organism-specific modulation of DNA repair outcomes may influence observed sgRNA efficiency.

While investigating parameters to optimize rates of homology-directed repair (HDR) during genome editing experiments, we found that the frequency of error-prone repair outcomes also tended to increase when single-stranded HDR donor DNA was present in the editing reaction^9^. Prompted by this observation, we undertook a systematic exploration of the parameters underlying DNA-mediated stimulation of error-prone repair events. To avoid confounding effects stemming from the use of plasmid or other nucleic acid-mediated delivery of Cas9, we performed editing experiments using nucleofection to directly introduce a ribonucleoprotein complex (RNP) of Cas9 complexed with sgRNA into cells^3^,^6^,^9^. Here we show that the addition of non-homologous single-stranded DNA during Cas9-mediated gene targeting greatly increases the frequency of disrupting mutations in multiple human cell lines. Consequently, this dramatically increases the number of cells with homozygous gene disruptions within the edited population. Non-homologous DNA appears to drive cells towards error-prone instead of error-free repair pathways, thereby increasing the frequency of sequence disruption during genome editing.

## Results

### Complex nucleic acid enhances sequence disruption

**Figure 1:**
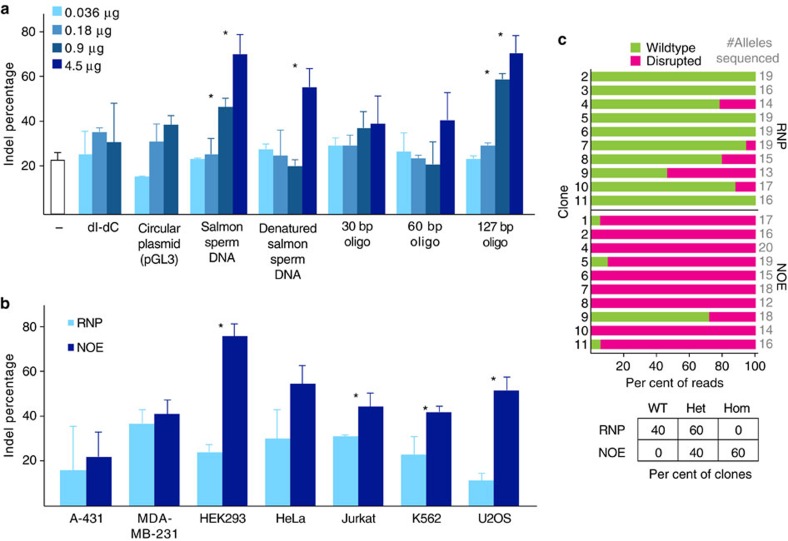
Non-homologous DNA increases gene disruption in multiple cell types. (**a**) Single- and double-stranded linear non-homologous DNA stimulates indel formation in HEK293T cells. Cas9 was targeted to the EMX1 locus with or without nucleic acid carrier agents (−, no nucleic acid). Indel formation was measured using a T7 endonuclease I assay and is presented as the mean±s.d. of at least two independent experiments (see Supplementary Data 1 for uncropped gels). * denotes significant difference between sample and no nucleic acid (−) control (*P*<0.05, Welch's *t*-test). (**b**) Non-homologous single-stranded DNA treatment (NOE) increases editing rates in multiple cell types. Editing was performed as described in panel A in multiple cell types either with (dark blue bars, NOE) or without (light blue bars, RNP) 4.5 μg of N-oligo. (**c**) NOE increases the frequency of homozygous gene disruption. HEK293T cells edited in panel (**b**) were clonally isolated and amplicons were sequenced to determine genotype. Each horizontal bar represents a single clone with green (wildtype sequence) or magenta (mutations disrupting EMX1) divisions sized according to the percentage of sequencing reads in each category. Zygosity is summarized in the lower table.

Targeting the EMX1 locus, we selected a sub-optimal RNP whose activity was ∼20% in HEK293T cells. We found that the addition of a 127-mer single-stranded DNA oligonucleotide derived from BFP, which lacks homology to the targeted locus and whose sequence is absent in the human genome, markedly increased the appearance of insertions and deletions (indels), as measured by a T7E1 assay (Fig. 1a). The ability of non-homologous oligonucleotides to increase editing efficiency was titratable and depended upon oligonucleotide length, with shorter oligonucleotides losing efficacy, potentially due to intracellular degradation. Native and denatured salmon sperm DNA were also capable of stimulating indels to a similar extent as synthetic single-stranded oligonucleotides, demonstrating that nucleotide sequence was not important for the effect. Neither heparin (multiple negative charges), spermidine (multiple positive charges), nor Poly deoxyinosinic-deoxycytidylic acid (dI-dC) had much effect on editing, implying that complex nucleic acid was necessary for stimulation (Supplementary Fig. 1). Free DNA ends were also required, as closed circular plasmid was ineffective (Fig. 1). We henceforth refer to the use of a non-homologous oligonucleotide to stimulate sequence disruption in concert with Cas9 RNP editing as ‘Non-homologous oligonucleotide enhancement' or ‘NOE'

We asked whether NOE was generalizable to multiple cell types, genomic loci and Cas9 delivery methods. We found that NOE stimulated indel formation in five out of the seven cell lines tested, with tissue types ranging from bone to blood, including a fivefold increase in indels in U2OS cells (Fig. 1b). We also observed NOE stimulation at the YOD1 and JOSD1 loci. This stimulation at the YOD1 locus ‘rescued' an otherwise completely ineffective guide, increasing the rate of indel formation from nearly undetectable to ∼17% (Supplementary Fig. 2A). NOE may be specific to RNP delivery, as it increased editing frequency at the AAVS1 locus in K562 cells together with electroporated Cas9 RNP but not when introduced with Cas9 and sgRNA plasmids (Supplementary Fig. 2B). We therefore focused our attention on NOE effects in conjunction with RNP editing.

### NOE increases homozygous gene disruption

Because the T7E1 indel formation assay operates on an edited pool of cells and does not report on individual alleles, we used TA/TOPO cloning and Sanger sequencing to determine if increased indel frequency corresponded to a higher number of clonal homozygous knockouts. We focused on HEK293T cells, which have a tetraploid genome and are thus a stringent test case for the formation of homozygous knockouts. Characterizing clonally isolated cells, we found that editing with RNP alone yielded 40% heterozygous clones and no homozygous knockouts, whereas NOE yielded 40% heterozygotes and 60% homozygous knockouts (Fig. 1c). Hence, NOE is a simple and effective technique to increase the frequency of homozygous gene disruption.

**Figure 2:**
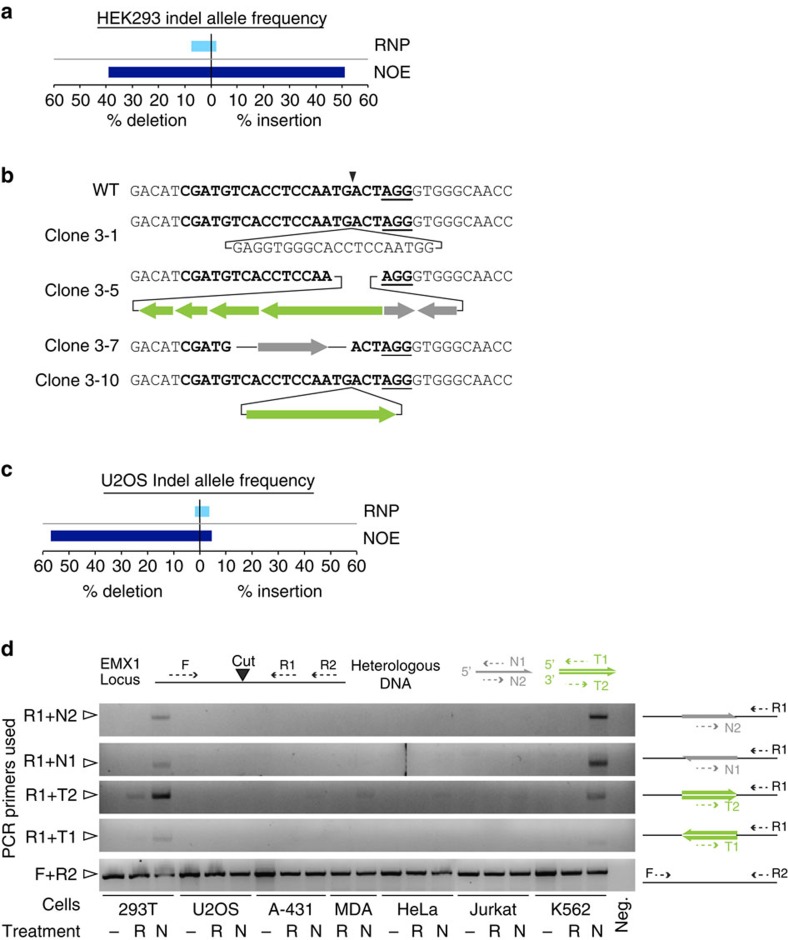
NOE promotes error-prone DNA repair events that differ between cell types. (**a**) NOE stimulates insertions and deletions in HEK293T cells. The allele frequency of deletions (left of plot) and insertions (right of plot) are shown for nucleofections performed with (dark blue, NOE) or without (light blue, RNP) N-oligo. Editing is summarized for each clone in Supplementary Fig. 3. Raw data is available in Supplementary Data 3. (**b**) Inserted sequences are derived from single- and double-stranded heterologous DNA. Wildtype EMX1 sequence is presented at top with the protospacer (bold), PAM (bold underline), and cut site (triangle) diagrammed. Four example alleles are presented below with sgRNA template (green) and/or non-homologous oligonucleotide (grey) sequence inserted in both orientations. Complete sequencing alignments are available in Supplementary Data 3. (**c**) NOE stimulates deletions in U2OS cells. Multiple sequence reads from edited cell populations are presented as described in Fig. 2a. (**d**) Insertion of non-homologous DNA primarily occurs in HEK293T and K562 cells. DNA harvested from unedited (-), RNP treated (R), or NOE treated (N) cell populations was evaluated using a panel of PCR reactions (diagrammed at top). Primers N1 and N2 anneal to the N-oligo sequence; primers T1 and T2 anneal to residual sgRNA template. Open arrow—position of the 700 bp ladder band.

Sequence analysis of the alleles in HEK293T editing reactions revealed that NOE increased the rate of both insertions and deletions relative to RNP treatment alone (Fig. 2a). These indels included deletions around the cut site and insertion of apparently random sequences, but surprisingly also occasional insertion of the non-homologous oligonucleotide and frequent insertion of the double-stranded DNA template used for *in vitro* transcription of the sgRNA (for example indels, Fig. 2b; all indels Supplementary Fig. 3). The frequent presence of double stranded sgRNA template sequence was particularly striking, as the non-homologous single stranded oligonucleotide was ∼1000-fold more abundant in the editing reactions (Supplementary Fig. 4). Notably, the sgRNA template was included in all editing reactions, but was only frequently observed at editing sites in conjuction with NOE. The occasional insertion of non-homologous DNA into DBSs has been reported in yeast^10^,^11^,^12^ and mice^13^,^14^,^15^ and the insertion of short phosphorothioate-protected oligonucleotides forms the basis of the GUIDE-Seq method to detect off-target genome editing events^16^, but the ability of non-homologous single-stranded DNA to stimulate DNA integration events is surprising and to the best of our knowledge unprecedented.

### The molecular outcome of NOE is cell line specific

Our observations of sequence insertion in HEK293T cells motivated us to investigate the molecular outcomes of NOE-mediated gene disruption in other cell types. Surprisingly, Sanger sequencing of editing in clonally isolated U2OS cells revealed that NOE primarily stimulated the appearance of large deletions, and not insertions, as compared with RNP alone editing (Fig. 2c). To determine the propensity of various cell types to insert sequences into a Cas9 break we developed a polymerase chain reaction (PCR) assay to amplify even rare integration of the non-homologous oligonucleotide or sgRNA transcription template in various orientations. This assay confirmed sequence insertion in K562 and HEK293T cells, but not in other cell lines that exhibited robust NOE stimulation (Figs 2d and 1b).

### Duplex non-homologous DNA is inserted into Cas9 breaks

Given the large excess of non-homologous single-stranded oligonucleotide over double-stranded sgRNA template during NOE, we asked if providing high concentrations of double-stranded non-homologous DNA (also derived from BFP) would also effectively stimulate sequence insertion in the cell lines that integrate exogenous nucleic acid. We tested both single- and double-stranded non-homologous DNA for their potential to increase indels at the EMX1 locus. To ensure that the double-stranded sgRNA template was completely removed, we treated the *in vitro* transcribed sgRNAs twice with DNAse before performing edits (see Methods). We found that using double-stranded DNA during NOE also stimulated indels, though about two-fold less effectively than single-stranded oligonucleotide (Supplementary Fig. 5A). Using a PCR assay for sequence integration, we found no evidence of sgRNA template insertion in samples treated to remove the template, but greater integration of the duplex DNA relative to single-stranded oligonucleotide (Supplementary Fig. 5B). This result suggests that explicitly designing a duplex DNA for integration could further bias cells towards a desired outcome, for example, inserting a cassette that encodes stop codons in multiple frames and orientations may bias cells towards knockout even more effectively than random indels. This strategy has been proposed for HDR-mediated gene disruption, but to our knowledge has not been attempted with NOE-mediated non-homologous integration of oligonucleotides^17^.

### NOE is consistent at on- and off-target sites

**Figure 3:**
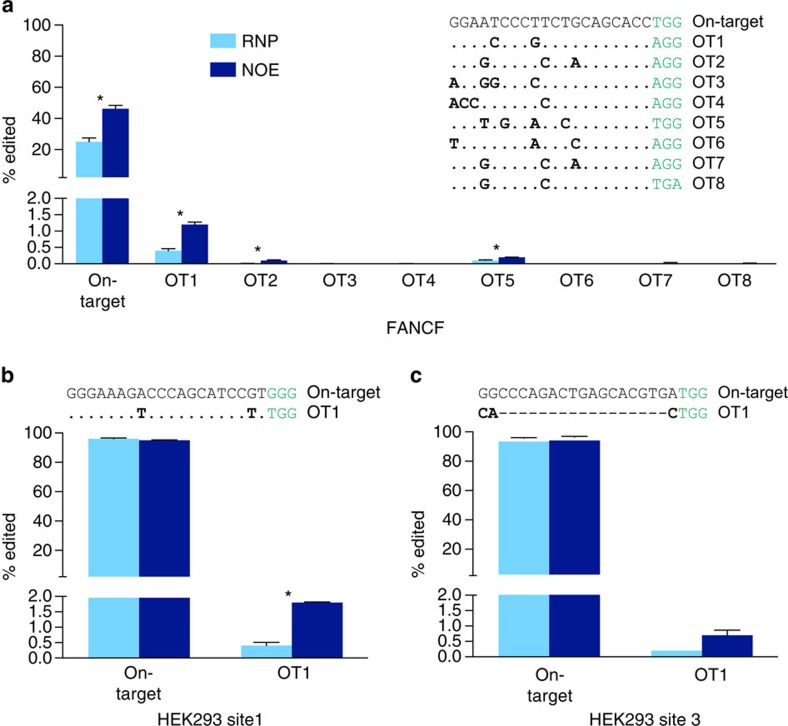
NOE is locus-independent. (**a**) NOE causes similar fold changes of editing at FANCF on-target and three known off-target sites^16^ in HEK293T cells. Cas9 was targeted to the FANCF locus with (dark blue, NOE) or without (light blue, RNP) non-homologous oligonucleotide and editing rates at FANCF or previously reported off-target (OT) loci were measured by NGS analysis. Data presented are the mean±s.d. of two biological replicates. *—denotes significant (*P*<0.05, Welch's *t*-test) difference between RNP and NOE samples. Full editing rates are presented in Supplementary Fig. 7. (**b**,**c**) NOE increases editing rates ∼2.9-fold at the HEK293 site 1 and HEK293 site 3 known off-target sites, but cannot increase editing at the already saturated on-target site. Data presented as described in panel (**a**).

NOE is a simple and effective way to boost gene disruption events at targeted loci and in multiple cell types, but off-target editing is a concern for targeted nucleases and treatments that preferentially mutagenize off-target sites would be undesirable. To investigate the effects of NOE at off-target sites, we performed editing reactions with and without NOE using sgRNAs whose off targets have been previously determined by the GUIDE-seq unbiased capture approach^16^. We used next generation sequencing to exhaustively monitor editing rates at the target site as well as representative, characterized off-target sites spanning a wide range of off-target editing rates. In the absence of NOE we observed editing at 5/10 GUIDE-seq identified off-target sites, and 7/10 off-target sites in conjunction with NOE. Consistent with a locus-independent effect, we found that NOE increased editing rates at both on-target and off-target sites in HEK293T and U2OS cells (Fig. 3 and Supplementary Figs 6 and 7). However, the magnitude by which NOE increased gene disruption at off-target sites does not differ significantly from the magnitude at on-target sites (mean, 2.8±1.0 fold on target versus 2.9±0.9 fold off target, Supplementary Fig. 7) in the HEK293T cell background. For example, editing at the FANCF on-target site increased from ∼25% to ∼46% with NOE, while editing at OT5 increased from 0.1% to 0.2% (Fig. 3). Saturating levels of on-target editing in the U2OS cell line prevented analysis of fold effects for on-target editing, but we observed similar fold changes compared to HEK293T cells at the off-target sites (Supplementary Fig. 6, RNP samples). We conclude that NOE generally stimulates error-prone repair independent of locus. We hypothesize that NOE could be especially effective when paired with strategies that increase the specificity of Cas9 cutting though this remains to be tested^18^,^19^.

## Discussion

Our data reveal that non-homologous single-stranded oligonucleotides delivered simultaneously with Cas9 RNPs stimulate error-prone DNA repair, greatly increasing genomic disruption frequency during genome editing experiments. This approach, which we term ‘NOE', for ‘Non-homologous oligonucleotide enhancement', allows one to rescue underperforming guide RNAs and more readily obtain homozygously edited cell clones.

Taken together, our data support a model in which cells fidelitously repair most Cas9-generated DSBs using error-free repair pathways that do not produce measurable indels. Subsequent rebinding and cutting of a fidelitously repaired locus by Cas9 then propagates a cycle of cutting and repair that terminates when infrequent error-prone repair causes indels that ablate portions of the Cas9 protospacer and/or PAM. This would thereby prevent further cutting and produce a measurable genomic outcome (Supplementary Fig. 8). We note that if repair outcomes depend upon cellular context, this could explain why Cas9–sgRNA combinations with high *in vitro* activity can exhibit poor cellular activity and why sgRNA activity differs between cell lines (Fig. 1b)^7^,^8^.

We hypothesize that NOE induces a cellular response that increases the frequency of error-prone repair events and thereby could divert the fidelitous cutting/repair cycle towards measurable genomic disruption. Differences in repair pathway utilization between cell types could explain how NOE increases gene disruption in multiple cell types but with distinct molecular outcomes. For example, a preference for alternative end-joining in HEK293T cells as opposed to a preference for non-homologous end-joining in U2OS cells could explain why NOE stimulates insertions in HEK293Ts but deletions in U2OS. Thus, understanding the NOE mechanism may provide key insights into how human cells decide between various DNA repair pathways. In the short term, we anticipate that NOE will be valuable to researchers generating homozygous gene disruption cell lines or organisms, and could increase signal-to-noise during high-throughput, arrayed gene editing screens.

## Methods

### Cell lines and cell culture

A-431, HEK293T, HeLa, Jurkat, K562, MDA-MB-231 and U2OS cells were acquired from the UC Berkeley Cell Culture Facility. Cells were tested for mycoplasma contamination before use. A-431, HeLa and MDA-MB-231 cells were maintained in DMEM glutamax medium supplemented with 10% fetal bovine serum, 1% sodium pyruvate, 1% non-essential amino acids and 100 μg ml^−1^ penicillin–streptomycin. HEK293T and U2OS cells were maintained in DMEM medium supplemented with 10% fetal bovine serum, 1% sodium pyruvate and 100 μg ml^−1^ penicillin–streptomycin. Jurkat and K562 cells were maintained in RPMI medium supplemented with 10% fetal bovine serum, 1% sodium pyruvate, and 100 μg ml^−1^ penicillin–streptomycin.

Clonal cells were generated by dilution cloning using a Combidrop (Thermo Scientific, Waltham, MA).

### Cas9 and RNA preparation

*Streptococcus pyogenes* Cas9 (pMJ915, Addgene #69090) with two nuclear localization signal peptides and an HA tag at the C terminus was expressed in Rosetta2 DE3 (UC Berkeley Macrolab) cells. Cell pellets were sonicated, clarified, Ni2+-affinity purified (HisTraps, GE life sciences), TEV cleaved, cation-exhanged (HiTrap SP HP, GE life sciences), size excluded (Sephacryl S-200, GE life sciences) and eluted at 40 μM in 20 mM HEPES KOH pH 7.5, 5% glycerol, 150 mM KCl, 1 mM dithiothreitol (DTT)^20^. sgRNAs were generated by HiScribe (NEB E2050S) T7 *in vitro* transcription using PCR-generated DNA as a template (dx.doi.org/10.17504/protocols.io.dm749m). Complete sequences for all sgRNA templates can be found in Supplementary Data 2.

### Cas9 RNP assembly and nucleofection

100 pmoles of Cas9-2NLS was diluted to a final volume of 5 μl with Cas9 buffer (20 mM HEPES (pH 7.5), 150 mM KCl, 1 mM MgCl_2_, 10% glycerol and 1 mM TCEP) and mixed slowly into 5 μl of Cas9 buffer containing 120 pmoles of L2 sgRNA. The resulting mixture was incubated for 10 min at room temperature to allow RNP formation. 2E+05 cells were harvested, washed once in PBS, and resuspended in 20 μl of nucleofection buffer (Lonza, Basel, Switzerland). 10 μl of RNP mixture, 4.5 μl of N-oligo, and cell suspension were combined in a Lonza 4d strip nucleocuvette. Reaction mixtures were electroporated, incubated in the nucleocuvette at room temperature for 10 min, and transferred to culture dishes containing pre-warmed media (dx.doi.org/10.17504/protocols.io.dm649d). Editing outcomes were measured 2 days post nucleofection by T7E1 (see below). Resuspension buffer and electroporation contions were the following for each cell line: A-431 in SF with EQ-100, HEK293T in SF with DS-150, HeLa in SE with CN-114, Jurkat in SE with CL-120, K562 in SF with FF-120, MDA-MB-231 in SE with CH-125 and U2OS in SE with CM104.

### Transfection

An AAVS1 guide (GTGTCCCTAGTGGCCCCACTG) was introduced into the PX330 (Addgene #42230) all-in-one Cas9/sgRNA vector. Approximately 1 μg of this plasmid was transfected into 200,000 K562 cells using Lipofectamine LTX (Thermo Fisher, Waltham, MA). 100 pmoles of N-oligo (oCR283), if used, was mixed with plasmid before packaging into liposomes.

### PCR amplification of edited regions

PCR amplification of EMX1 was done using primers oCR295 and oCR296. PCR amplification of YOD1 was done using YOD1f and YOD1r. PCR amplification of JOSD1 was done using JOSD1f and JOSD1r. PCR amplification of AAVS-1 was done using oCR142 and oCR143. PCR amplification of FANCF_1 was done using oGJR146 and oGJR159. PCR amplification of FANCF_2 was done using oGJR147 and oGJR160. PCR amplification of FANCF_3 was done using oGJR148 and oGJR161. PCR amplification of FANCF_4 was done using oGJR149 and oGJR162. PCR amplification of FANCF_5 was done using oGJR150 and oGJR163. PCR amplification of FANCF_6 was done using oGJR151 and oGJR164. PCR amplification of FANCF_7 was done using oGJR152 and oGJR165. PCR amplification of FANCF_8 was done using oGJR153 and oGJR166. PCR amplification of FANCF_9 was done using oGJR154 and oGJR167. PCR amplification of HEK293-1-1 was done using oGJR155 and oGJR168. PCR amplification of HEK293-1-2 was done using oGJR156 and oGJR169. PCR amplification of HEK293-3-1 was done using oGJR157 and oGJR170. PCR amplification of HEK293-3-2 was done using oGJR158 and oGJR171. PCR reactions were performed with 200 ng of genomic DNA and Kapa Hot Start high-fidelity polymerase with the GC buffer. The thermocycler was set for one cycle of 95 °C for 5 min, 30 cycles of 98 °C for 20 s, 62 °C for 15 s, 72 °C for 30 s and one cycle of 72 °C for 1 min, and held at 4 °C.

### T7EI assay

The rate of Cas9-mediated gene disruption was measured by T7 endonulcease I digestion of hybridized PCR products. PCR DNA (200 ng) in 1 × NEB Buffer 2 was hybridized in a thermocycler under the following conditions: 95 °C for 5 min, 95–85 °C at −2 °C s^−1^, 85–25 °C at −1 °C s^−1^, and held at 4 °C. Ten units of T7EI (NEB, M0302) were added to the sample and was incubated at 37 °C for 15 min. The sample was then immediately run on a 2% agarose gel containing ethidium bromide. Band intensities were quantified by imageJ. Indel percentage was calculated using the following equation: (1-(1-(cut product intensities/uncut+cut product intensities))^1/2^) × 100 (ref. 5).

### Sanger sequencing of edited loci

PCRs from pooled or clonal cell populations were TA-cloned (Zero Blunt TOPO, Thermo Scientific, Waltham, MA) to recover individual amplicons. At least 12 amplicons were sequenced from each clone or pool and mapped against predicted amplicon sequence using Geneious (Biomatters, Auckland, New Zealand). Aligned reads were manually scored as ‘deletions'—reads missing predicted sequence, or ‘indels'—reads with additional sequence inserted. The metric ‘Per cent of Reads' was calculated as (# reads containing deletions+# reads containing insertions)/total reads. %Deletion (# reads containing deletions/total reads) and %Indel (# reads containing InDels/total reads) were also calculated. Full sequences are available in Supplementary Data 3.

### Insert-based PCR assay

To assay the insertion of N-oligo and sgRNA template DNA into the cut site, a reverse primer (oGJR102) was designed to pair with forward primers homologous to the BFP N-oligo inserted in the forward direction (oGJR097), BFP N-oligo inserted in the reverse direction (oGJR098), the T7 promoter of the sgRNA template inserted in the forward direction (oGJR099) and the T7 promoter of the sgRNA template inserted in the reverse direction (oGJR100). Presence of EMX1 DNA in the PCR reaction was verified using the EMX1 PCR performed above. PCR reactions were performed with 200 ng of genomic DNA and Kapa Hot Start high-fidelity polymerase. The thermocycler was set for one cycle of 95 °C for 5 min, 30 cycles of 98 °C for 20 s, 64 °C for 15 s, 72 °C for 30 s and one cycle of 72 °C for 1 min, and held at 4 °C. The sample was then run on a 2% agarose gel containing ethidium bromide.

### Next-generation sequencing (NGS) analysis of edited cells

PCR amplicons were repaired, A-tailed, adapter ligated and amplified using the NEB (Ipswich, MA) Next Ultra kit (NEB E7370L). Dual indexing (NEB E7600S) was implemented to permit multiplex sequencing. All samples were pooled in equimolar amounts and sequenced on an Illumina (San Diego, CA) MiSeq using the MiSeq Reagent Kit v3 (2 × 300). The ends of reads were trimmed until encountering a window of 30 bases with average quality score >30, using the programme sickle (https://github.com/najoshi/sickle). Trimmed reads were then aligned to the predicted amplicon sequence using Bowtie2 (http://bowtie-bio.sourceforge.net/bowtie2/index.shtml) and were classified as edited if the alignment contained an indel within an eight-basepair window around the cut site or unedited otherwise.

### qPCR on RNP

sgRNA template and N-oligo DNA present in the nucleofection reaction mixture was quantified on a Mastercycler 2 qPCR machine (Eppendorf, Hamburg). Primers oCR427 and oCR428, sgRNA template; oGJR103 and oGJR104, N-oligo; (Supplementary Data 2) were used at a final concentration of 500 nM in Power SYBR green reaction mixture (Thermo Fisher). Reaction conditions were 95 °C for 10 min followed by 40 cycles of 95 °C for 30 s and 65 °C for 60 s. The ratio of N-oligo to sgRNA template was quantified using the equation 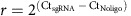. Ratios from three serial dilutions of template DNA were averaged and presented as mean±s.d.

### Statistical testing

Pairwise comparisons between experimental and control samples were made using Welch's *t*-test.

### Data avaliability

Sanger sequencing data is available in Supplementary Data 3. NGS sequencing data is available in the NIH Sequence Read Archive (BioProject ID PRJNA326133). All other data is available upon request.

## Additional information

**Accession codes:** Next Generation sequencing data is available in the NIH Sequence Read Archive under accession code: BioProject ID PRJNA326133.

**How to cite this article:** Richardson, C.D. *et al*. Non-homologous DNA increases gene disruption efficiency by altering DNA repair outcomes. *Nat. Commun.* 7:12463 doi: 10.1038/ncomms12463 (2016).

## Supplementary Material

Supplementary Information Supplementary Figures 1-8

Supplementary Data 1 Uncropped gels from Figures 1A, 1B, 2D, and Supplementary Figure 6.

Supplementary Data 2 Sequences of PCR primers, sgRNA templates, etc

Supplementary Data 3 Zip archive containing FASTA alignments used to generate Figure 2 and Supplementary Figure 3
