# Supplementary material for: Non-homologous DNA increases gene disruption efficiency by altering DNA repair outcomes
Source: Nat Commun. 2016 Aug 17;7:12463. doi: 10.1038/ncomms12463 (PMC4992056; doi:10.1038/ncomms12463)
Supplement: Supplementary Information — Supplementary Figures 1-8 [file ncomms12463-s1.pdf]

Supplementary Figure 1

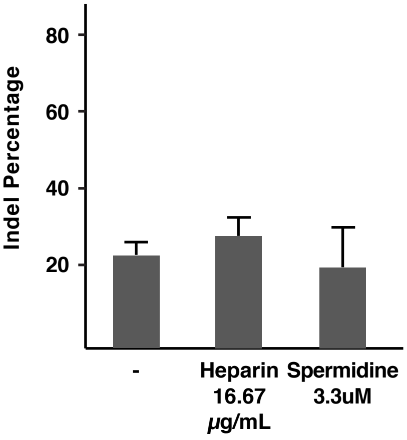

Supplementary Figure 1: Heparin and spermidine do not stimulate editing at the EMX1 locus in HEK293T cells. Editing was performed as described in Figure 1A.

Supplementary Figure 2

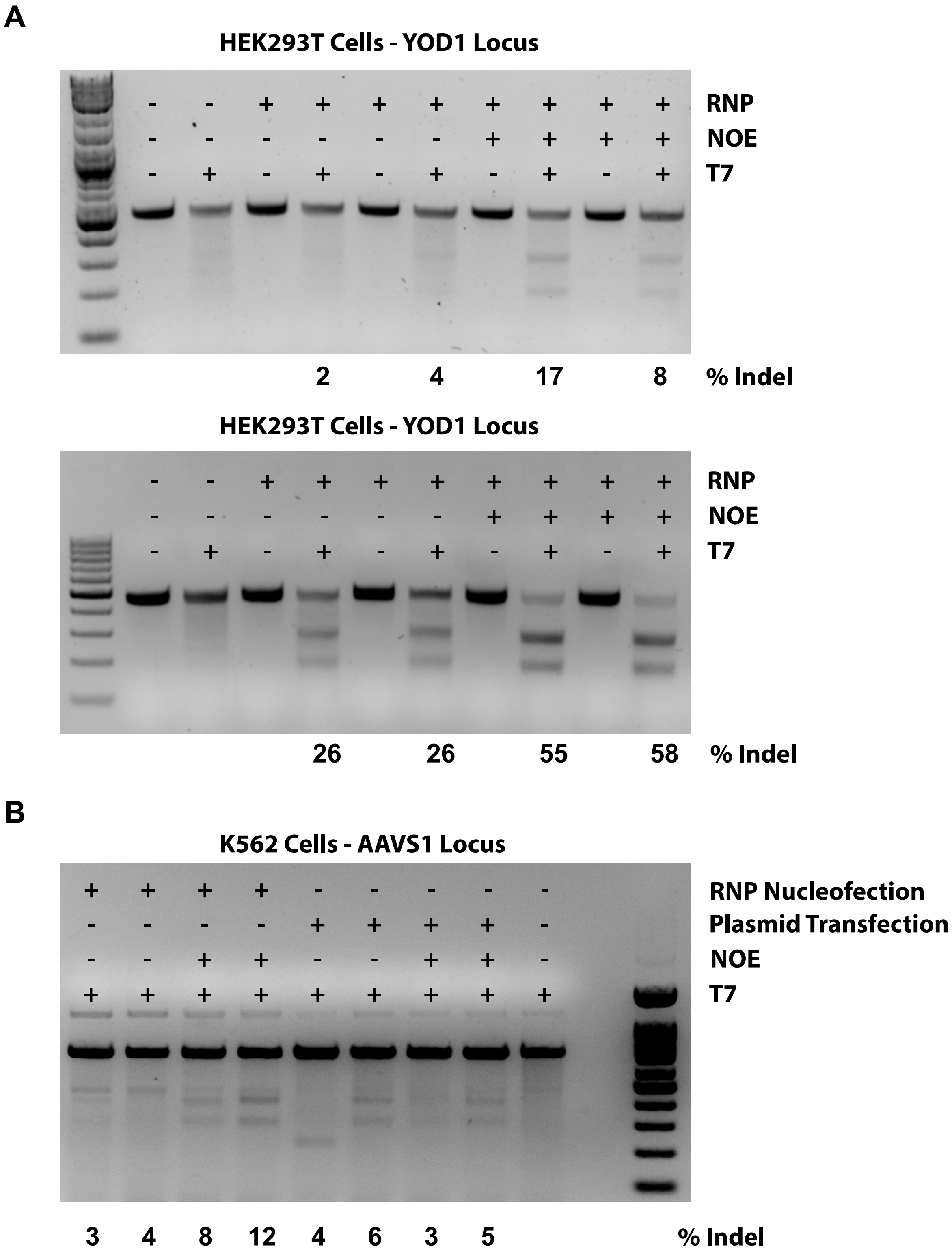

Supplementary Figure 2: (A) NOE at the YOD1 and JOSD1 loci in HEK293T cells. Editing experiments were performed with (+ NOE) or without (-NOE) N-oligo as indicated. Indel percentage was assayed by T7 endonuclease cleavage and gel densitometry. (B) NOE occurs at the AAVS1 locus in K562 cells during RNP nucleofection, but not plasmid transfection experiments. Data presented as described in panel (A)

Supplementary Figure 3

	Clone	WT	Deletions	Insertions
HEK293 RNP	2	(19)		
	3	(16)		
	4	(11)		9bp/2bp template indel (3)
	5	(19)		
	6	(19)		
	7	(18)	216bp del (1)	
	8	(12)	21bp del (1),<br>295bp del (1),<br>433bp del (1)	
	9	(7)	3bp del (6)	
	10	(15)	7bp del (1),<br>21bp del (1)	
	11	(16)		
HEK293 NOE	1	(1)	3bp del (3),<br>5bp del (1)	3bp ins (2),<br>20bp unknown ins (10)
	2		15bp del (11)	5bp/26bp indel (5)
	4		7bp del (20)	
	5	(2)	5bp del (7)	11bp/5bp indel (2),<br>339bp/5bp template/N-oligo indel (8)
	6		10bp del (7)	251bp template ins (8)
	7			86bp/14bp N-oligo indel (10)<br>20bp/1bp indel (8)
	8		8bp del (2)	7bp ins (10)
	9	(13)		10bp/4bp indel (5)
	10		19bp del (7)	119bp template ins (7)
	11	(1)	16bp del (6)	82bp/18bp N-oligo indel (8),<br>55bp/20bp N-oligo indel (1)

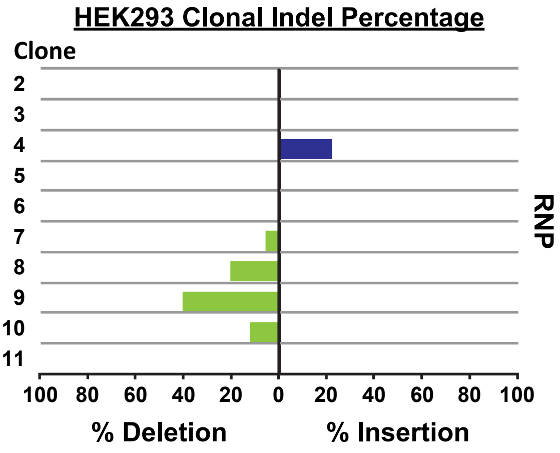

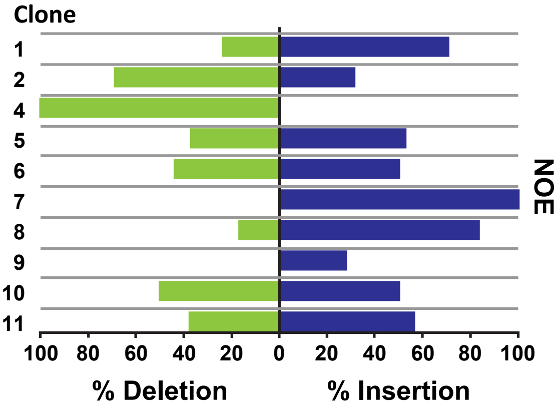

	Treatment	WT	Deletions	Insertions
U2OS	RNP	(56)	186bp del (1)	5bp/71bp indel (1),<br>20bp/3bp indel (1)
	NOE	(9)	1-10bp del (8),<br>20-60bp del (5)	8bp ins (1)

Supplementary Figure 3: NOE stimulates insertions and deletions in HEK293T cells. Sequence reads from clonal cell populations were binned into three categories (WT, unmodified; deletions, clear removal of sequence; and insertions, added sequence with or without flanking deletions) and presented in table form. The complete sequence of each read can be found in Document S3. Bar graphs present clonal indel percentages.

Supplementary Figure 4

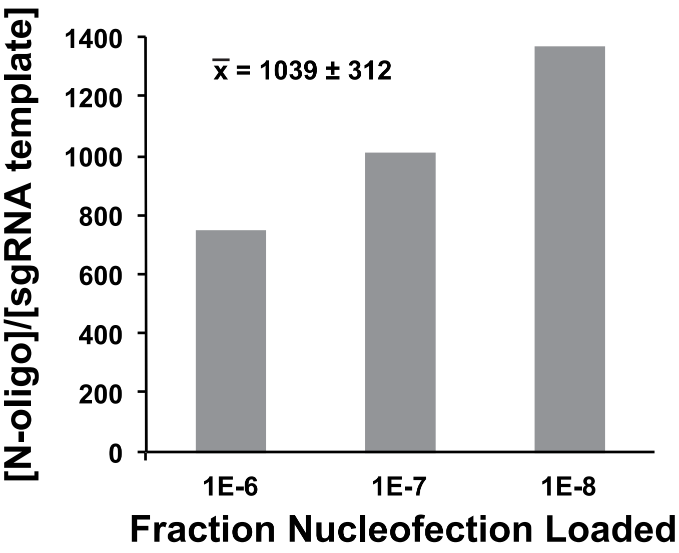

Supplementary Figure 4: N-oligo is present in excess of sgRNA template. Nucleofection mixtures were serially diluted and the abundance of N-oligo or sgRNA template were quantified by qPCR. Fold enrichment of N-oligo over sgRNA template are presented for three serial dilutions of nucleofection mixtures. Inset number is the mean +/- standard deviation of these three values.

Supplementary Figure 5

A

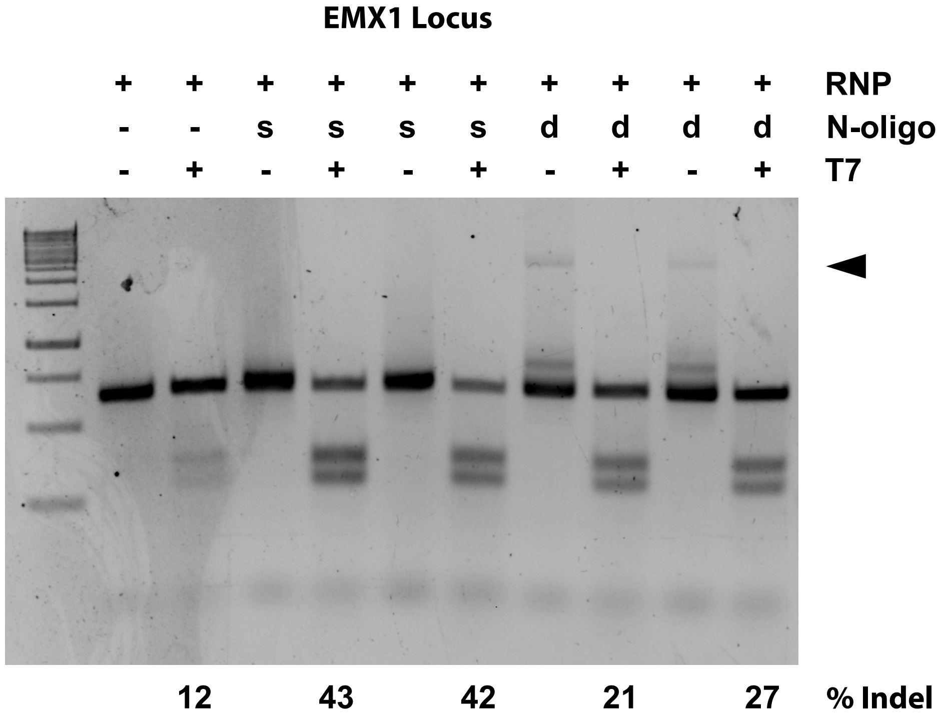

B

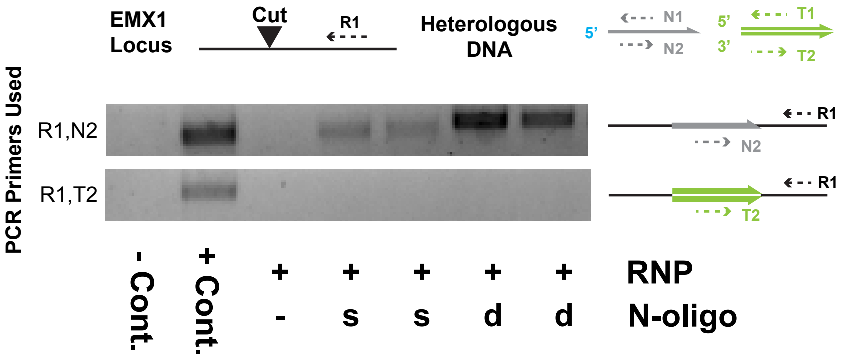

Supplementary Figure 5: sgRNA template DNA is not required for NOE. (A) Extensively purified sgRNA (free of sgRNA template DNA) was used in editing experiments at the EMX1 locus. T7 editing rates increased dramatically with the addition of single or double stranded DNA. (B) PCR for N-oligo or sgRNA template insertion indicates that insertion events are derived from the N-oligo rather than the sgRNA template. Double stranded N-oligos (d) tend to insert more efficiently than single stranded N-oligos (s).

Supplementary Figure 6

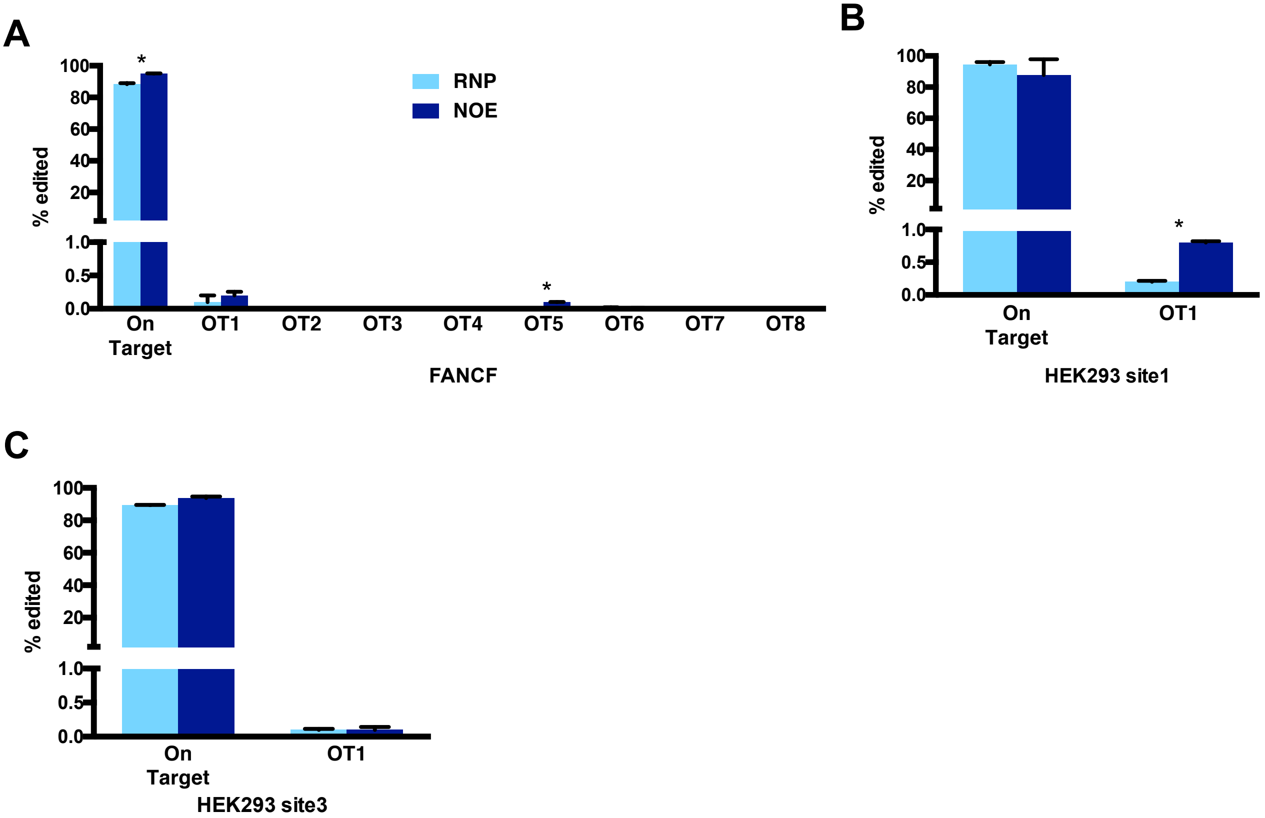

Supplementary Figure 6: NOE consistently stimulates gene disruption at on- and off-target sites. (A) N-oligo significantly increases fold changes of editing at FANCF on-target and two known off target sites[16] in U2OS cells. The magnitude of the N-oligo effect is suppressed at the FANCF on-target site, as gene disruption in the (-) N-oligo sample is near saturation. (B)-(C) N-oligo increases editing rates approximately 2.9-fold at the HEK293 site 1 off-target site, but cannot increase editing at the already saturated on-target sites. Data presented as described in **Figure 3**.

Supplementary Figure 7

Cell Type	Guide	Amplicon	% Edit	NOE % Edit	Fold Effect Each	Fold Effect Mean	
HEK293T	EMX1	On Target*	24.13±0.03%	76.00±0.05%	3.1		On Target
	YOD1	On Target*	3.00±1.41%	12.50±6.36%	4.2		
	JOSD1	On Target*	26.00±0.00%	56.50±1.41%	2.2	2.8±1.0	
HEK293T	FANCF	On Target*	24.99±2.47%	46.25±2.10%	1.9		
		OT1*	0.39±0.06%	1.19±0.07%	3.0		p=0.45
		OT2*	0.02±0.01%	0.05±0.01%	1.9		
		OT3	0.01±0.00%	0.02±0.00%			
		OT4	0.01±0.00%	0.01±0.00%			
		OT5*	0.06±0.02%	0.17±0.00%	2.6	2.9±0.9	
		OT6	0.00±0.00%	0.02±0.00%			Off Target
		OT7	0.01±0.00%	0.04±0.03%			
		OT8	0.00±0.00%	0.04±0.02%			
HEK293T	HEK293 site 1	HEK1-1	95.89±0.52%	95.11±0.21%			
		HEK1-2*	0.44±0.10%	1.82±0.02%	4.1		
HEK293T	HEK293 site 3	HEK3-1	93.37±0.42%	94.14±0.63%			
		HEK3-2	0.19±0.00%	0.71±0.16%			
U2OS	FANCF	On Target*	88.37±0.61%	95.06±0.09%			
		OT1	0.05±0.05%	0.22±0.05%			
		OT2	0.01±0.00%	0.01±0.00%			
		OT3	0.00±0.00%	0.00±0.00%			
		OT4	0.00±0.00%	0.01±0.00%			
		OT5*	0.03%	0.05±0.00%			
		OT6	0.03±0.02%	0.02±0.01%			
		OT7	0.01±0.00%	0.01±0.00%			
		OT8	0.00±0.00%	0.00±0.00%			
U2OS	HEK293 site 1	HEK1-1	94.51±1.54%	87.70±10.2%			
		HEK1-2*	0.20±0.01%	0.79±0.02%			
U2OS	HEK293 site 3	HEK3-1	89.42±0.13%	93.80±0.89%			
		HEK3-2	0.05±0.01%	0.13±0.04%			

Supplementary Figure 7: Raw data used to generate bar graphs in Figure 3 and Extended Data Figure 6.

Supplementary Figure 8

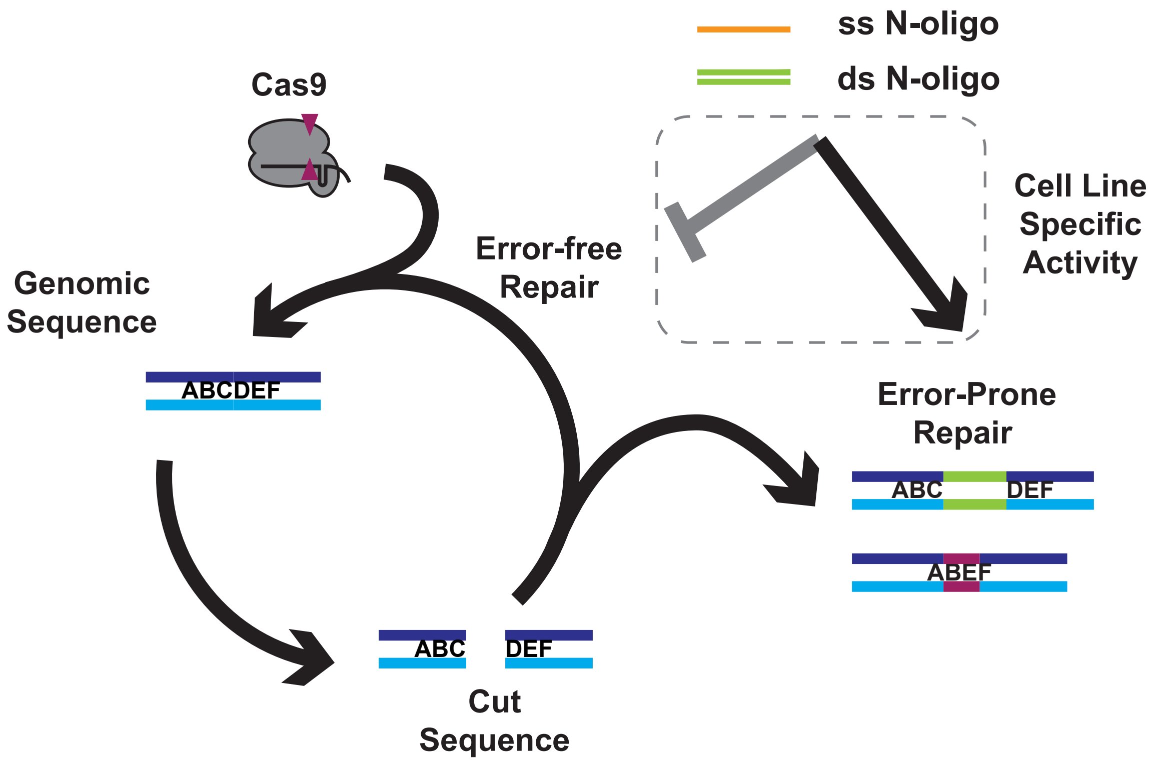

Supplementary Figure 8: Model for NOE. Cas9-sgRNA (grey) recognizes and cuts genomic sequence (ABCDEF). Cellular repair processes reseal most breaks in an error-free fashion, which restores the Cas9 recognition sequence and permits additional rounds of cutting. Error-prone repair events such as insertions (green sequence) and deletions (maroon region) disrupt the Cas9 recognition sequence and prevent cutting. Single and double stranded N-oligo act to inhibit error-free repair and promote error-prone repair events. NOE is cell-line dependent.
