# Supplementary material for: Non-homologous DNA increases gene disruption efficiency by altering DNA repair outcomes
Source: Nat Commun. 2016 Aug 17;7:12463. doi: 10.1038/ncomms12463 (PMC4992056; doi:10.1038/ncomms12463)

Figure 1A Gels

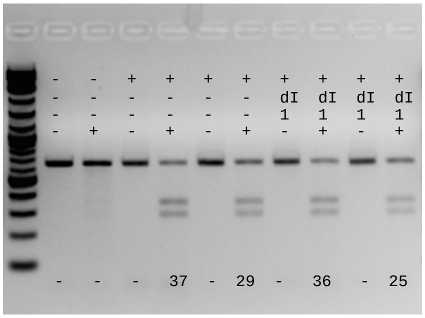

dI-dC = dI
Salmon Sperm = SS
Heparin = H
Spermadine = S
plasmid pgL3 = p
127bp ss BFP oligo = b1
120 bp ss BFP oligo = b2

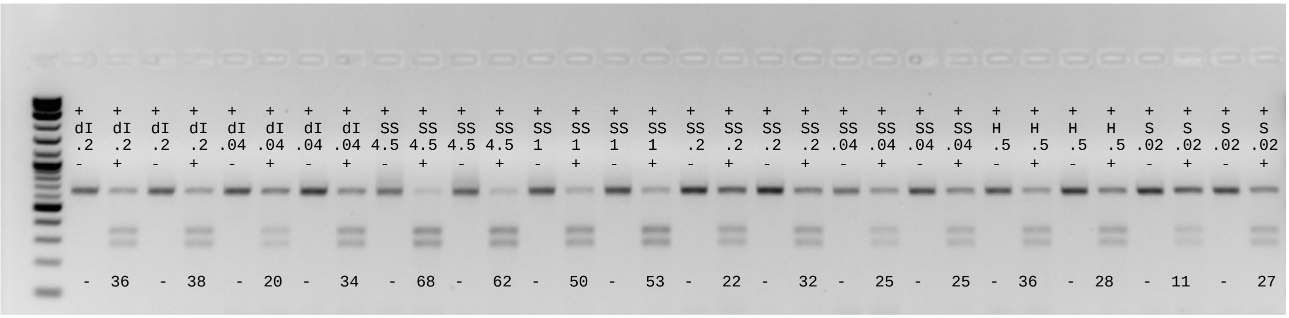

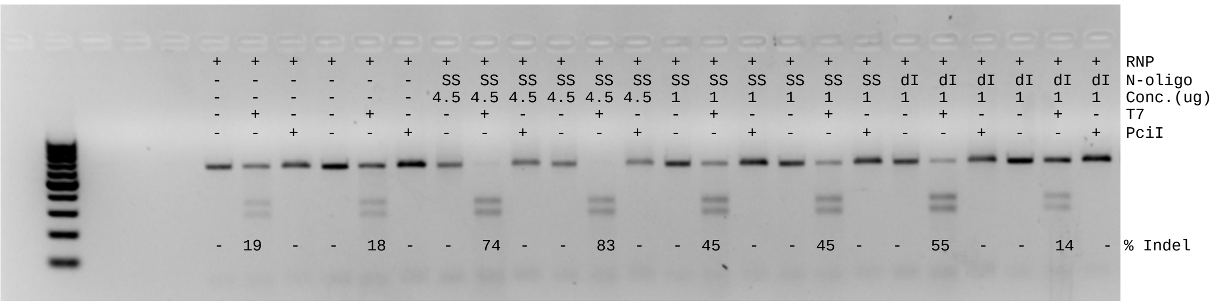

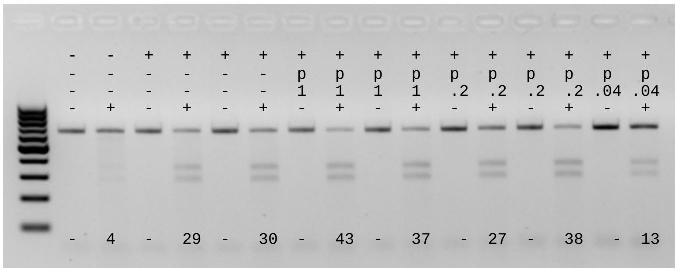

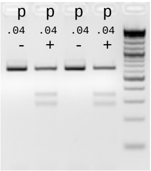

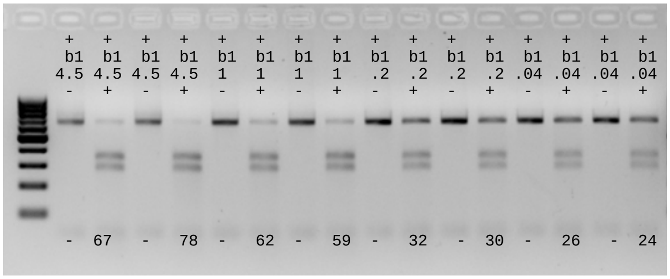

Figures 1A+1B Gels

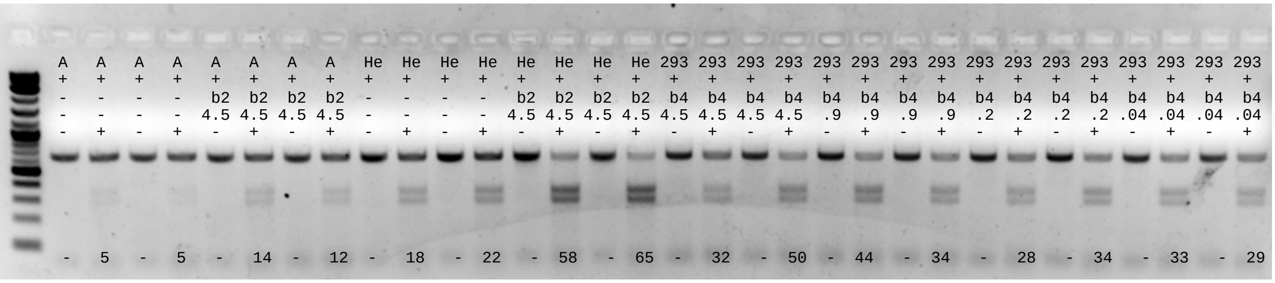

Cell Type	A	A	A	A	A	A	A	A	He	He	He	He	He	He	He	He	293	293	293	293	293	293	293	293	293	293	293	293	293	293	293	293
RNP	+	+	+	+	+	+	+	+	+	+	+	+	+	+	+	+	+	+	+	+	+	+	+	+	+	+	+	+	+	+	+	+
N-oligo	-	-	-	-	b2	b2	b2	b2	-	-	-	-	b2	b2	b2	b2	b4	b4	b4	b4	b4	b4	b4	b4	b4	b4	b4	b4	b4	b4	b4	b4
Conc.(ug)	-	-	-	-	4.5	4.5	4.5	4.5	-	-	-	-	4.5	4.5	4.5	4.5	4.5	4.5	4.5	4.5	.9	.9	.9	.9	.2	.2	.2	.2	.04	.04	.04	.04
T7	-	+	-	+	-	+	-	+	-	+	-	+	-	+	-	+	-	+	-	+	-	+	-	+	-	+	-	+	-	+	-	+
% Indel	-	5	-	5	-	14	-	12	-	18	-	22	-	58	-	65	-	32	-	50	-	44	-	34	-	28	-	34	-	33	-	29

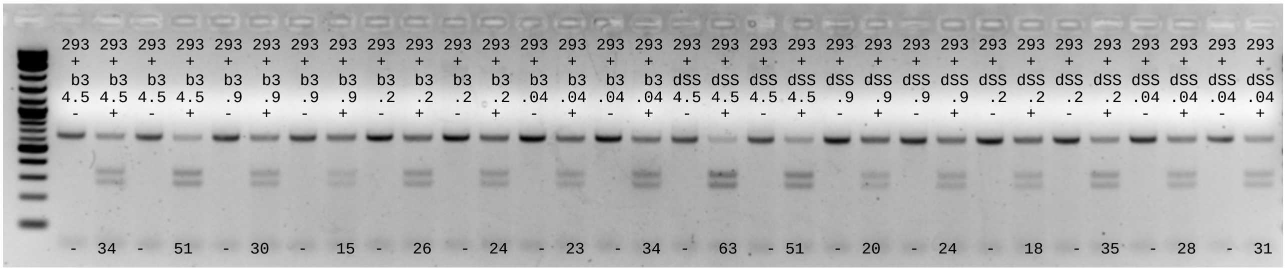

Cell Type	293	293	293	293	293	293	293	293	293	293	293	293	293	293	293	293	293	293	293	293	293	293	293	293	293	293	293	293	293	293	293	293
RNP	+	+	+	+	+	+	+	+	+	+	+	+	+	+	+	+	+	+	+	+	+	+	+	+	+	+	+	+	+	+	+	+
N-oligo	b3	b3	b3	b3	b3	b3	b3	b3	b3	b3	b3	b3	b3	b3	b3	b3	dSS	dSS	dSS	dSS	dSS	dSS	dSS	dSS	dSS	dSS	dSS	dSS	dSS	dSS	dSS	dSS
Conc.(ug)	4.5	4.5	4.5	4.5	.9	.9	.9	.9	.2	.2	.2	.2	.04	.04	.04	.04	4.5	4.5	4.5	4.5	.9	.9	.9	.9	.2	.2	.2	.2	.04	.04	.04	.04
T7	-	+	-	+	-	+	-	+	-	+	-	+	-	+	-	+	-	+	-	+	-	+	-	+	-	+	-	+	-	+	-	+
% Indel	-	34	-	51	-	30	-	15	-	26	-	24	-	23	-	34	-	63	-	51	-	20	-	24	-	18	-	35	-	28	-	31

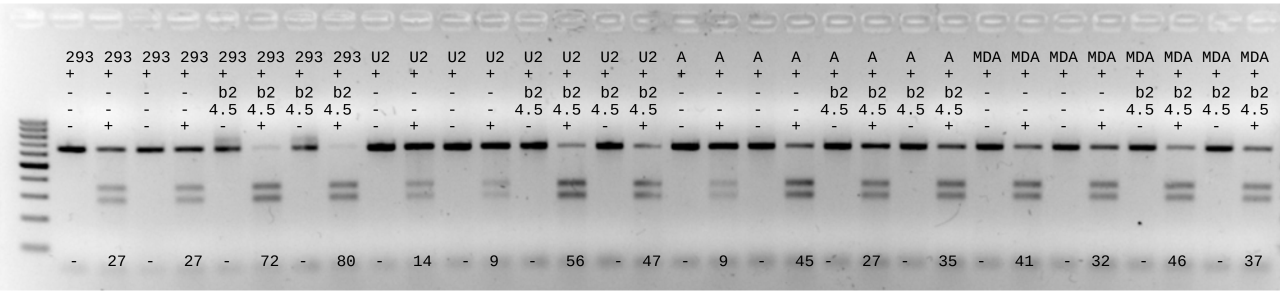

Cell Type	293	293	293	293	293	293	293	293	U2	U2	U2	U2	U2	U2	U2	U2	A	A	A	A	A	A	A	A	MDA	MDA	MDA	MDA	MDA	MDA	MDA	MDA
RNP	+	+	+	+	+	+	+	+	+	+	+	+	+	+	+	+	+	+	+	+	+	+	+	+	+	+	+	+	+	+	+	+
N-oligo	-	-	-	-	b2	b2	b2	b2	-	-	-	-	b2	b2	b2	b2	-	-	-	-	b2	b2	b2	b2	-	-	-	-	b2	b2	b2	b2
Conc.(ug)	-	-	-	-	4.5	4.5	4.5	4.5	-	-	-	-	4.5	4.5	4.5	4.5	-	-	-	-	4.5	4.5	4.5	4.5	-	-	-	-	4.5	4.5	4.5	4.5
T7	-	+	-	+	-	+	-	+	-	+	-	+	-	+	-	+	-	+	-	+	-	+	-	+	-	+	-	+	-	+	-	+
% Indel	-	27	-	27	-	72	-	80	-	14	-	9	-	56	-	47	-	9	-	45	-	27	-	35	-	41	-	32	-	46	-	37

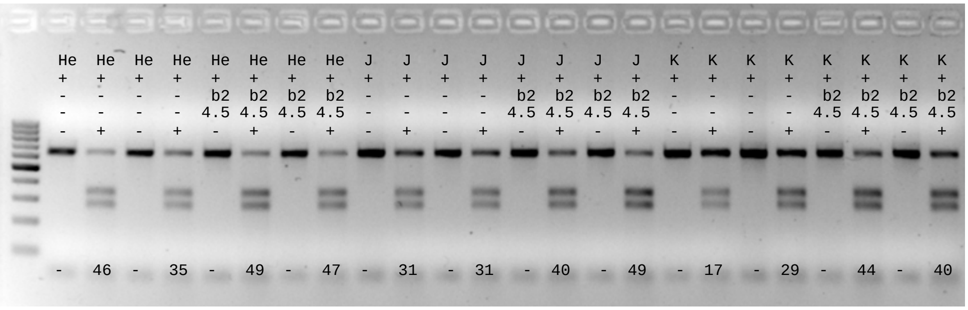

Cell Type	He	He	He	He	He	He	He	He	J	J	J	J	J	J	J	J	K	K	K	K	K	K	K	K
RNP	+	+	+	+	+	+	+	+	+	+	+	+	+	+	+	+	+	+	+	+	+	+	+	+
N-oligo	-	-	-	-	b2	b2	b2	b2	-	-	-	-	b2	b2	b2	b2	-	-	-	-	b2	b2	b2	b2
Conc.(ug)	-	-	-	-	4.5	4.5	4.5	4.5	-	-	-	-	4.5	4.5	4.5	4.5	-	-	-	-	4.5	4.5	4.5	4.5
T7	-	+	-	+	-	+	-	+	-	+	-	+	-	+	-	+	-	+	-	+	-	+	-	+
% Indel	-	46	-	35	-	49	-	47	-	31	-	31	-	40	-	49	-	17	-	29	-	44	-	40

dI-dC = dI
Salmon Sperm = SS
Heparin = H
Spermidine = S
plasmid pgL3 = p
127bp ss BFP oligo = b1
120bp ss BFP oligo = b2
60bp ss BFP oligo = b3
30bp ss BFP oligo = b4
denatured Salmon Sperm = dSS

HEK293 = 293
U2OS = U2
A-431 = A
MDA-MB-231 = MDA
HeLa = He
Jurkat = J
K562 = K

Figure 2D Gels

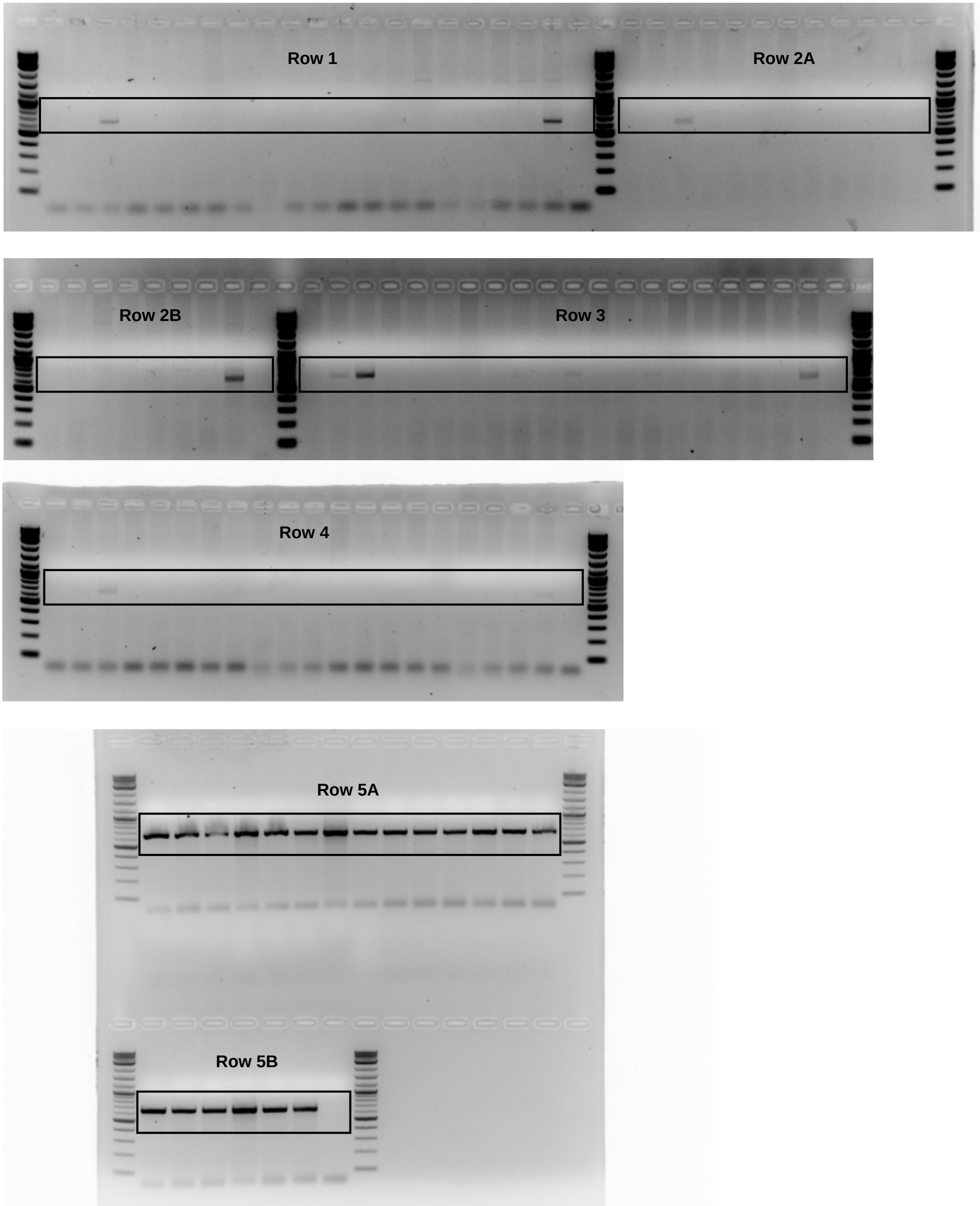

Extended Data Figure 6 Gels

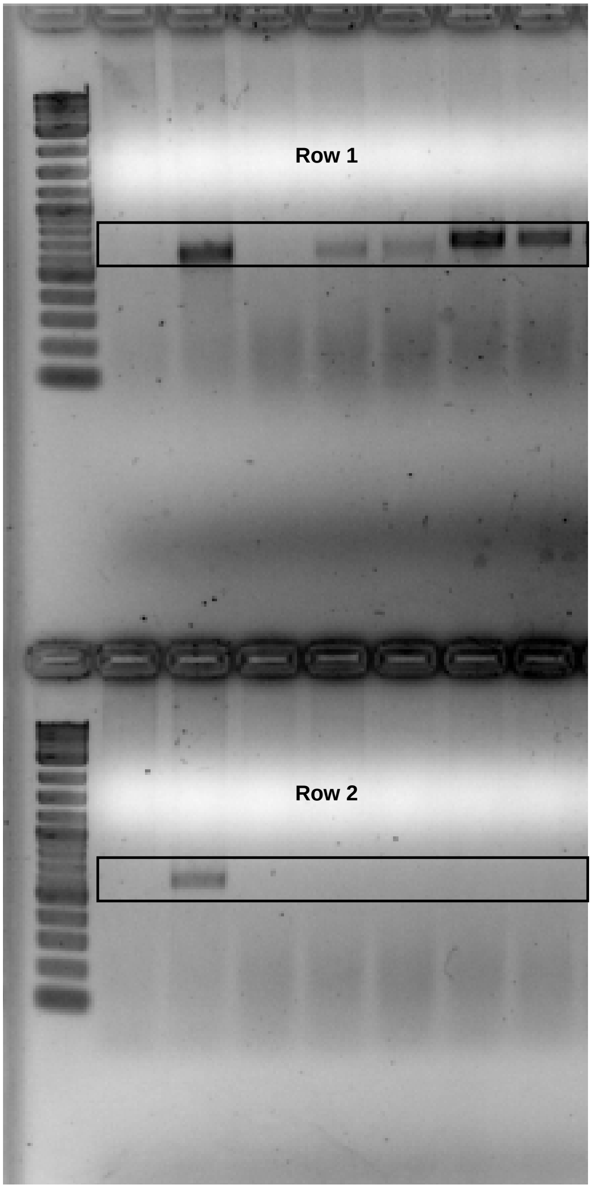

Supplement: Supplementary Data 1 — Uncropped gels from Figures 1A, 1B, 2D, and Supplementary Figure 6. [file ncomms12463-s2.pdf]
