# Supplementary material for: Non-homologous DNA increases gene disruption efficiency by altering DNA repair outcomes
Source: Nat Commun. 2016 Aug 17;7:12463. doi: 10.1038/ncomms12463 (PMC4992056; doi:10.1038/ncomms12463)
Supplement: Supplementary Data 2 — Sequences of PCR primers, sgRNA templates, etc [file ncomms12463-s3.docx]

**Supplementary Data 2**

**PCR Primers – insert PCR assay**

oGJR097 (N2) CAGTGCTTCAGCCGCTAC

oGJR098 (N1) GTAGCGGCTGAAGCACTG

oGJR099 (T2) GAGCTAGAAATAGCAAGTTAAAATAAGGC

oGJR100 (T1) GCCTTATTTTAACTTGCTATTTCTAGCTC

oGJR102 (R1) GTGGAGCTGGAGGTAGAGAC

oCR295 (F) GCCATCCCCTTCTGTGAATGTTAGAC

oCR296 (R2) GGAGATTGGAGACACGGAGAGCAG

**PCR Primers – amplification of edited regions**

oCR142 (AAVS1) GCGTCAGAGCAGCTCAGGTTCTG

oCR143 (AAVS1) CCCTTCTTGTAGGCCTGCATCATCA

oCR295 (EMX1) GCCATCCCCTTCTGTGAATGTTAGAC

oCR296 (EMX1) GGAGATTGGAGACACGGAGAGCAG

YOD1f (YOD1) CAGTGAACAAAGATTCTTACTGGT

YOD1r (YOD1) AAAATCGATATCTCTATTGCTCCT

oGJR051 (OT1) GGAAGTCTGGAACAGCTATGATGC

oGJR096 (OT1) GACTATTGGTAGCAGACATGTC

oGJR090 (OT2) GCACCCAATAGAGGAGTATGCAG

oGJR091 (OT2) CAGACACCAAATGAGTACAAGCCG

oGJR053 (OT3) GCTGGTGGGAAGTGTTTGGAG

oGJR071 (OT3) CTGTAGGATGCTGAGTTGGTCC

oGJR054 (OT4) ACACAACCAGCAGTGCAAGC

oGJR072 (OT4) GACGCAGGAATGCTACTAGG

JOSD1f (JOSD1) ATTCCAATCTTTTCTCCCAGTGA

JOSD1r (JOSD1) CCTGCTAACGCAATCTAGCT

oGJR146 (FANCF_1) AAAGCCGCCCTCTTGCCTC

oGJR159 (FANCF_1) CCAGAGTCAAGGAACACGGATAAAG

oGJR147 (FANCF_2) GGGCCAGTCCTTTGTAAGCATC

oGJR160 (FANCF_2) CTGGGTTTGGTTGGCTGCTC

oGJR148 (FANCF_3) GAAAGTGAACTGGTTTGGGCCTC

oGJR161 (FANCF_3) CTCCAAAGGGAATACAGCCCTG

oGJR149 (FANCF_4) ACGCGCTCGCTGGCATG

oGJR162 (FANCF_4) GAAGGCTGACCACGAGGTG

oGJR150 (FANCF_5) GCAAGCCCTGCCACTGTAG

oGJR163 (FANCF_5) CTGCCATGTGCCTTATCCTATTGTG

oGJR151 (FANCF_6) AGAGCGTCCGCAAACC

oGJR164 (FANCF_6) CCAGCTCCCTCCGTAGGTC

oGJR152 (FANCF_7) ATGGTCTGATCTTGGGCCTG

oGJR165 (FANCF_7) GCTACTGTGTTTCCTGTGTCCAAG

oGJR153 (FANCF_8) AACAGGAAGGTGAACTGGTTTG

oGJR166 (FANCF_8) GATTCCCTGCTAGAGTTCCAAAG

oGJR154 (FANCF_9) CTTCTTCTCATTGCTGTGCTTC

oGJR167 (FANCF_9) GCAGCGGCTCTCAGCAAG

oGJR155 (HEK293-1-1) GGAGAGGAGGGAGAGTCTGTC

oGJR168 (HEK293-1-1) CCTGAGTCAATGCAGATAGAGC

oGJR156 (HEK293-1-2) GGCACCAGCAGCAGCAATTAG

oGJR169 (HEK293-1-2) GAGGAGGTCCATCTGTCTGCATTTG

oGJR157 (HEK293-3-1) TTGGCATGAGAAACCTTGGAGAG

oGJR170 (HEK293-3-1) GGTCCCTCCTCTCCTGGTG

oGJR158 (HEK293-3-2) GCCAAAGGATCAACAGCCAATTC

oGJR171 (HEK293-3-2) GACTGTATCACTCCCGTGGAG

**qPCR Primers**

oCR427 (sgRNA tem) GGATCCTAATACGACTCACTATAGCGA

oCR428 (sgRNA tem) AAAAGCACCGACTCGGTGC

oGJR103 (N-oligo) CCTGAAGTTCATCTGCACCACC

oGJR104 (N-oligo) AAGTCGTGCTGCTTCATGTGG

**sgRNA T7 template DNA** (Protospacer in bold)

EMX1:

GGATCCTAATACGACTCACTATAGCGATGTCACCTCCAATGACTGTTTTAGAGCTAGAAATAGCAAGTTAAAATAAGGCTAGTCCGTTATCAACTTGAAAAAGTGGCACCGAGTCGGTGCTTTTTT

YOD1:

GGATCCTAATACGACTCACTATAGCATAGTACACACTAGTAAAGGTTTTAGAGCTAGAAATAGCAAGTTAAAATAAGGCTAGTCCGTTATCAACTTGAAAAAGTGGCACCGAGTCGGTGCTTTTTT

JOSD1:

| GGATCCTAATACGACTCACTATAGTGTCCTGGAAGACGTTATTGGTTTTAGAGCTAGAAATAGCAAGTTAAAATAAGGCTAGTCCGTTATCAACTTGAAAAAGTGGCACCGAGTCGGTGCTTTTTT  FANCF   \| GGATCCTAATACGACTCACTATAGGAATCCCTTCTGCAGCACCGTTTTAGAGCTAGAAATAGCAAGTTAAAATAAGGCTAGTCCGTTATCAACTTGAAAAAGTGGCACCGAGTCGGTGCTTTTTT  HEK293-1 \| \| --- \| \| GGATCCTAATACGACTCACTATAGGGAAAGACCCAGCATCCGTGTTTTAGAGCTAGAAATAGCAAGTTAAAATAAGGCTAGTCCGTTATCAACTTGAAAAAGTGGCACCGAGTCGGTGCTTTTTT  HEK293-3 \| \| GGATCCTAATACGACTCACTATAGGCCCAGACTGAGCACGTGAGTTTTAGAGCTAGAAATAGCAAGTTAAAATAAGGCTAGTCCGTTATCAACTTGAAAAAGTGGCACCGAGTCGGTGCTTTTTT \| \| AAVS1 \| \| GGATCCTAATACGACTCACTATAGTGTCCCTAGTGGCCCCACTGTTTTAGAGCTAGAAATAGCAAGTTAAAATAAGGCTAGTCCGTTATCAACTTGAAAAAGTGGCACCGAGTCGGTGCTTTTTT \| |
| --- | --- | --- | --- | --- | --- |

**N-oligo Sequence**

127 bp N-oligo (oCR283): TCATGTGGTCGGGGTAGCGGCTGAAGCACTGCACGCCGTACGTCAGGGTGGTCACGAGGGTGGGCCAGGGCACGGGCAGCTTGCCGGTGGTGCAGATGAACTTCAGGGTCAGCTTGCCGTAGGTGGC

120 bp N- oligo (oCR236): CCTGAAGTTCATCTGCACCACCGGCAAGCTGCCCGTGCCCTGGCCCACCCTCGTGACCACCCTGACGTACGGCGTGCAGTGCTTCAGCCGCTACCCCGACCACATGAAGCAGCACGACTT

60 bp N-oligo (oCR239):

CCCTCGTGACCACCCTGACGTACGGCGTGCAGTGCTTCAGCCGCTACCCCGACCACATGA

30 bp N-oligo (oGJR87):

CCCTCGTGACCACCCTGACGTACGGCGTGC

**Plasmids**

plasmid: pgL3 (Promega, Madison, WI)
