# Supplementary material for: Non-homologous DNA increases gene disruption efficiency by altering DNA repair outcomes
Source: Nat Commun. 2016 Aug 17;7:12463. doi: 10.1038/ncomms12463 (PMC4992056; doi:10.1038/ncomms12463)
Supplement: Supplementary Data 3 — Zip archive containing FASTA alignments used to generate Figure 2 and Supplementary Figure 3 [file ncomms12463-s4.zip › Readme.rtf]

This zip file contains aligned FASTA files generated from Sanger sequencing reactions performed on clonal edited HEK293 and pooled edited U2OS cells. HEK293 clones - RNP only editing1-2.fasta1-3.fasta1-4.fasta1-5.fasta1-6.fasta1-7.fasta1-8.fasta1-9.fasta1-10.fasta1-11.fastaHEK293 clones - RNP+N-oligo editing3-1.fasta3-2.fasta3-4.fasta3-5.fasta3-6.fasta3-7.fasta3-8.fasta3-9.fasta3-10.fasta3-11.fastaU2OS cells - RNP only editingU2OS-RNP.fastaU2OS cells - RNP+N-oligo editingU2OS-RNP-Carrier.fasta
